# Long-term experience with gene augmentation therapy in patients with inherited retinal disease associated with biallelic mutations in ***RPE65***

**DOI:** 10.1515/medgen-2024-2067

**Published:** 2025-02-12

**Authors:** Birgit Lorenz

**Affiliations:** c/o Justus-Liebig-University Giessen TransMIT Centre of Translational Ophthalmology Am Galgenberg 37 35321 Laubach Germany

**Keywords:** *RPE65* mutation-associated inherited retinal degeneration *RPE65*-IRD, gene augmentation therapy, voretigene neparvovec, functional results, retinal effects

## Abstract

*RPE65* biallelic mutation-associated inherited retinal degeneration (IRD) is currently the only IRD for which gene therapy is approved. This narrative review provides a brief overview of the disease and an update of the current literature on outcomes following the approval of treatment with voretigene neparvovec (LuxturnaTM) in 2017 (USA) and Europe (2018). Post-marketing results confirm a significant therapeutic effect of this gene augmentation on rod function similar to that seen in the phase 1 to 3 clinical trials. The full-field chromatic light sensitivity test is an appropriate test to demonstrate early and sustained effects of treatment. Visual acuity and visual fields may improve in less advanced disease. Accelerated chorioretinal atrophy (CRA) is a previously unrecognised adverse effect that is now reported in 13 % to 50 % of treated eyes. If central, visual acuity loss and paracentral visual field defects may occur. Further studies are needed to identify patients at risk of CRA in order to maximize patient benefit from a costly intervention.

## Introduction

*RPE65* biallelic mutation-associated inherited retinal degeneration (IRD) is currently the only IRD with an approved gene replacement therapy. It uses a specially designed adeno-associated virus 2 (AAV2) vector containing the normal RPE65 gene (voretigene neparvovec, VN, Luxturna™), which is delivered into the subretinal space after standard 3-port vitrectomy. The procedure usually follows the recommendations of Spark Therapeutics in the USA (https://sparktx.com/LUXTURNA_US_Prescribing_Information.pdf) and of Novartis Suisse which markets the drug outside the USA (https://www.novartis.com/sg-en/sites/novartis_sg/files/Luxturna-Oct2021.SIN-App200122.pdf). Typically, after appropriate dilution according to company instruction, 1.5 × 10^11^ vg (vector genome) in a total volume of 0.3 mL are injected through a 41 g cannula between outer retina and retinal pigment epithelium (RPE), resulting in a transient bleb equivalent to a localised retinal detachment lasting approximately 1 to 2 days. The German national ophthalmic societies have published their recommendations for the use of VN in 2019 (https://dog.org/wp-content/uploads/sites/11/2013/03/10.1007_s00347-019-0906-2.pdf). In addition to details of the surgical procedure and perioperative medication, patient selection based on the molecular genetically confirmed diagnosis and the status of the outer retina is discussed in detail. A prerequisite for VN to work is the preservation of photoreceptor cells and the underlying retinal pigment epithelium RPE in which the gene is expressed. The identification of the essential structures is based on appropriate optical coherence scans of the central retina. Since the approval of VN, the estimated number of treated patients has been at least 200 in the US and 200 in Europe (Claudio Spera, formerly Novartis Pharma Suisse, personal communication 2022). This narrative review provides a brief overview of the disease, the preclinical results and the clinical trials that led to the approval of the therapy. The narrative review of post-marketing data is based on a PubMed literature search, last accessed on 8 August 2024. It describes post-marketing functional and anatomical results and discusses them in terms of patient-relevant outcomes and personal expert experience.

## The disease

### Epidemiology and phenotypes

Biallelic mutations in *RPE65* cause a spectrum of autosomal recessive inherited retinal phenotypes, now termed* RPE65* mutation-associated IRD or *RPE65*-IRD. *RPE65*-IRD is extremely rare with an estimated prevalence of 1 in 300 000 births based on a number of different reports [27]. It includes Leber congenital amaurosis (LCA, about 5 % *RPE65*-IRD), early onset severe retinal dystrophy (EOSRD), and juvenile retinitis pigmentosa (about 1 % *RPE65*-IRD) [6, 30]. The spectrum of phenotypes parallels the severity of rod and cone dysfunction and degeneration respectively. Recently, a comprehensive review of the available literature including 100 relevant publications found a much higher variation of numbers within the RP and the LCA groups [39], which may indicate uncertainties in the reported epidemiological data.

**Table 1:** Characteristic retinal findings in *RPE65*-IRD and associated clinical signs

– Rod-cone degeneration with variable degree of cone dysfunction in the first decade of life
– absent rod ERG; residual or absent cone ERG [e. g. 22, 30]
– profound nyctalopia in all and dependence of visual performance on good lighting from birth regardless of severity of phenotype
– range of BCVA up to logMAR 0.0, typically 1.0 [6]
– sensory defect nystagmus ±
– Lack of blue light autofluorescence (BAF) in children and adolescents despite largely normal fundus [29]
– residual BAF in the presence of residual activity of the isomerohydrolase enzyme encoded by *RPE65* [28], sequence variants without loss of function (Lof) [45]
– BAF variable with increasing age, typically in patients diagnosed with retinitis pigmentosa [23]
– Optical coherence tomography of the retina (SD-OCT, SS-OCT): Ellipsoid zone (EZ) shows early on structural changes and foveal hypoplasia [23, 28]
– Late findings: salt and pepper fundus with or without bone spicules or large chorioretinal atrophies [33]

### Pathophysiology and clinical course

Table 1 lists the main characteristics of *RPE65*-IRDs. Visual acuity and visual fields are mostly measurable during the 1^st^ and 2^nd^ decade of life but blindness occurs without therapy in the 3^rd^ to 4^th^ decade of life [6, 16, 27, 33]. *RPE65* encodes an isomerohydrolase in the retinal pigment epithelium (RPE) that is essential for retinol recycling [35]. Some *RPE65* sequence variants result in residual enzyme activity and display a much later phenotype i. e. rod-cone dystrophy (RCD) with juvenile onset [15, 28]. In addition to profound night blindness due to the enzyme defect, lack of fundus autofluorescence under blue light is a hallmark of the disease in the early years when the retina may appear relatively unremarkable [29]. It is caused by reduced accumulation of lipofuscin in the RPE due to the enzymatic action of the enzyme, which results in little or no rhodopsin being present in the rod outer segments which are phagocytosed in the RPE.

### Molecular genetics and genotype-phenotype correlation

Although previous reports on the natural history of the disease have not described a clear genotype-phenotype correlation [6], a recent paper found a more severe phenotype for loss-of-function sequence variants compared to missense sequence variants associated with residual function of the isomerohydrolase [43], supporting previous reports [15, 28]. Sequence variants of unknown significance (VUS) pose a challenge in terms of patient selection for therapy. Reclassification of VUS has been based on functional studies, *in silico* models, case reports and familial segregation studies [47]. To demonstrate the biallelic presence of sequence variants in *RPE65*-IRDs, familial segregation studies are highly recommended, regardless of the type of sequence variants.

### The challenge of disease detection

Early detection of disease has become important to patients who may be eligible for treatment. Without appropriate clinical evaluation, including retinal imaging, electrodiagnostics and psychophysics, there is a high risk that milder phenotypes may not be diagnosed as IRD, as early fundus changes may be very subtle, leading to suspicion of infantile or neurological nystagmus or central visual impairment of unclear origin. A strong suspicion for *RPE65*-IRD is the strong dependence of visual performance on adequate illumination. The rate of undiagnosed RPE65-IRD has decreased significantly since the pharmaceutical industry and patient organisations such as ProRetina Deutschland have made significant efforts to raise awareness of the disease among healthcare providers. In the survey conducted by the European Vision Institute Clinical Research Net (EVICR.net), the percentage of central vision impairment has decreased from 20 % in 2019 as initial misdiagnosis to 6 % in 2021 [27]. However, the proportion of undiagnosed cases may still be considerably high outside specialized centers, as for example in Germany, the percentage of patients with IRDs who receive a molecular genetic diagnosis is much lower in non-university institutions than in specialized ophthalmologic centers [21].

## Preclinical studies and clinical trials prior to approval

Breakthrough results in the long-term restoration of rod and cone vision by single-dose recombinant adeno-associated virus (rAAV)-mediated gene transfer to the retina in a canine model of RPE65-IRD [1] have paved the way for human application. Successful phase 1–3 clinical trials led to an effective adenovirus-associated (AAV) vector-based approach (AAV2.hRPE65v2, voretigene neparvovec (VN)) with a single subretinal injection [38]. The results were promising, as the majority of patients experienced a significant increase in visual performance at reduced light levels, indicating improved rod vision, while changes in visual acuity representing cone function did not reach statistical significance. A requirement for approval of the novel therapy by the Food and Drug Administration (FDA) was the demonstration of a patient-relevant benefit. Therefore, the Multi-Luminance Mobility Test (MLMT) was developed [6], which documented significantly improved outcomes after VN therapy in the absence of significant changes in best-corrected visual acuity, the classic outcome measure used to demonstrate therapeutic efficacy. The FDA approved VN (Luxturna™) in the US in 2017, and the European Medicines Agency (EMA) in Europe in 2018. Since then, many other countries have approved this gene augmentation therapy. A recent review looked at the available literature from 1974 to 2021 that examined the episomal persistence of different rAAV vector genomes and the preclinical and clinical evidence of long-term effects of different RPE65 gene replacement therapies [24]. Viral genomes were reported as transcriptionally active episomes for at least 22 months, the longest follow-up in the study. In dogs with *RPE65*-IRD, treatment effects lasted for almost a decade and were more pronounced the earlier the intervention. In humans with *RPE65*-IRD, long-term persistence of therapeutic effects has been reported of up to 5 years (MLMT) and 7.5 years (FST).

## Postmarketing results

The number of VN therapy results reported has increased since its approval. Table 2 provides an overview of the 13 papers with at least 4 treated patients based on a literature search of PubMed (last search August 8, 2024). Data include: patient demographics (number of patients, age range, follow-up), functional data (best corrected visual acuity and full-field light stimulus threshold at baseline and at follow-up as well as data on visual field changes) and morphological data from multimodal retinal imaging. Some patients are included in > 1 paper as indicated in the table entry. The number of patients/eyes and the methods used at baseline and follow-up often varied considerably making statistical conclusions difficult. This problem is typical of post-marketing data that do not follow a strict protocol of predefined outcome measures. This is also true for the PERCEIVE registry (EUPAS31153, http://www.encepp.eu/encepp/viewResource.htm?id=37005). From the data available to date, a clear correlation of the functional and morphological outcomes with the bleb location is not seen, although some reports have found a trend (for more details see Table 2). One reason may be that the location of the bleb, i.e. the area between the neuroretina and the underlying RPE where VN is applied during surgery (usually a single 0.3 mL bleb) cannot be reliably predicted. This is due to many factors such as variable adhesion of the degenerated neuroretina to the underlying RPE, internal structure of the retina etc. Some surgeons therefore create several smaller blebs depending on the intraoperative situation, or perform limited peeling of the internal limiting membrane to facilitate bleb formation [10]. An additional problem is that the original bleb may move to the periphery due to air-fluid exchange at the end of the surgery [11].

### Best corrected visual acuity (BCVA)

In most patients, the median or mean BCVA did not change significantly (Table 2). This is consistent with the pre-marketing results. However, individual patients experienced significant (at least 0.3 logMAR) changes for better or worse. Paediatric eyes generally had a higher chance of improvement [14, 26, 43]. In some cases, a decrease in BCVA correlated with worsening of retinal changes seen on multimodal retinal imaging (colour and infrared fundus photography, blue-light fundus autofluorescence (BAF), spectral domain optical coherence tomography (SD-OCT), after VN. These changes may be associated with vitritis, which may indicate inflammation or an unwanted immune response. The exact aetiologies are controversial.

**Table 2:** Comparison of post-marketing reports on VN treatment of *RPE65*-IRD involving at least 4 patients

Author Study design Diagnosis	n Patients age range (y)	Follow-up	BCVA at BL	BCVA at FU	FST at BL	FST at FU	Additional findings reported
[41]§ single centre EOSRD	5, 14–36	3 mo	FC to 0.2 (dec)	Improved or stable	Blue – 3.39 dB Red 0.83 dB	Age strong predictor for gain; ped> adults	DAC cyan improved
[40] multicentre	41, 2–44	Mean 10 mo (0.25 to 18.5)	Pediatric mean 20/150 (FC to 20/40) Adult mean 20/260 (LP to 20/70).	75 % ± 1 line change; no age effect; BL and CRA without effect on outcome	white 0.6 dB ± 3.7	Mean improvement 21.1 dB ± 16.6	Mean CFT pediatric 210 µm, adult 176 µm; mild thinning at FU regardless age Foveal detachment ± did not influence outcome
[13] multicentre (4 sites)	10, 5 – 20	Mean 11.3 mo (4–18).	logMAR 0.82 ± 0.51	Change logMAR 0.09 ± 0.45	White −1.3 log cd.s/m^2^ ±: 0.44	Mean improvement −3.21 log cd.s/m^2^	All with CRA from a larger cohort 8/10 bilateral; within bleb 38.9 %; within/outside bleb 55.5 %; outside bleb 5.5 %;growth of atrophy over time 100 %; mean myopia – 5.7 dpt (range −11.5 to +1.75) Visual field: paracentral scotoma related to atrophy 3 eyes, unrelated to atrophy 3 eyes (100 % of overall visual field, 13 eyes).
[9] single centre LCA	14, 4–17	Median 513 days (167 – 766)	Mean 20/191 logMAR 0.98 (0.4 – 1.7	logMAR −0.8 (0.10 to 1.60)	White −2.0 log cd.s/m^2^ ±: 0.7	White −4.1 log cd.s/m^2^ ±0.9	mean CST (19 eyes) at BL 215 µm (192 – 247); at FU 206 µm (185 to 230) GVF III4e (13 eyes): At BL mean 163 sum degrees (0 to 767). At FU 384 sum degrees (17 to 1047)
[14] single centre LCA	4, 3–6	Mean 18.5 mo	logMAR 1.3 – 0.7	Mean improvement-0.31 logMAR 4-year-old patient: 1 eye from 1.3 to 0.6 6-year-old patient: 1 eye from 0.7 to 0.0	White – 9.5 dB (1 patient)	Improved by ≥ 30 dB at 6 months	BAF absent, nystagmus +, improved post VN; mobility test failed < 40 lux pre and passed at 4 lux post VN; ERG scotopic and flicker non-recordable preop, flicker positive in 2 patients post VN; measurable Goldmann isopter III4e in 3 patients post VN.
[18] Single centre	12, 4–26	4 – to 15 mo	BCVA given in letters, Snellen, LP, HM	No significant change	White Range −1.137 to −2.455 cd.s/m^2^	White Range −1.319 to −5.565 cd/m^2^	All Danish patients treated with VN Vitritis minimal to mild 9/23 eyes, 4 with outer retinal changes and subsequent new CRA; median interval to inflammation 89 days
[44] single centre	6, 7–17	6 mo	Mean 20/100 logMAR 0.7 ± 0.08	mean improvement -0.2 logMAR ± 0.7	NA	NA	reduced CRT, reduced central ONL thickness compared to age-matched healthy eyes
[41]# 2 centres*	38, 2–44	≥ 12 mo	logMAR 1.1 ± 0.64 n=67	logMAR change 0.04 ± 0.53 (n=67)	White −24.91dB ± 1.74 n=48	Change −15.99 ± 11.43 n=27	Correlation CRA – change FST CRA (n = 20) −22.78 ± 9.21 (n=15) No CRA (n = 51) −7.51 ± 7.70 (n=12)
[12]* multicentre (15 countries LCA, EOSRD; RP	103, 2–51	Mean 0.8 y ± 0.64; max 2.3 y	logMAR 1.14 ± 0.57 (n = 148)	logMAR change -0.03 ± 0.55 (2 years, n = 24)	White – 4.56 dB ± 10.88 (127 eyes)	White At 12 mo (n = 42): -18.24 dB ± 14.62	Mean CRT 209.2 µm ± 45.82 (117 eyes); Any ocular AEs 17.5 % o
[19] RP (4), LCA (2)	6, 18–49	8.2 mo (1–12)	logMAR 1.28 ± 0.71	logMAR 1.46 ± 0.6	White −4.41 dB ±10.62	White −11.98 dB ±113.83	Retinal atrophy: 12/12 eyes injection site 8/12 within bleb area (mild, asymmetrical) 2/12 within bleb and peripheral, severe
[20] single centre EOSRD	4, 12–37	Mean 22.3 mo	ND	ND	Blue – 2.4 cd/m^2^ ± 2.1 (6 eyes)	Blue – 4.03 cd/m^2^ ± 1.15 (6 eyes)	DA-2CTP results correspond to FST improvement plus spatial resolution
[2]$ multicentre (5)	14, 5–26	2.2 y_ ± 0.8	logMAR mean 0.8	ND	White Mean – 1.9 cd/m^2^	ND	Total eyes treated 187 = 14.4 % with atrophy CRA touchdown 14 eyes, nummular 15 eyes perifoveal 12 eyes; > 1 type of atrophy 15 eyes: growth rates touchdown < nummular < perifoveal (16.7 _ 1.8 mm^2^/year)
[26] single centre EOSRD, RP	19, 8–40 5 pediatric 14 adult	Median 15.1 mo (1.1–32.2) Pediatric 15.5 mo (1.1–21.4) Adult 13.9 mo (6.2 – 32.2)	logMAR 1.25 (0.2–2.3) Pediatric: 0.55 (0.3–1.5) Adult: 1.4 (0.2–2.3)	Median BCVA stable (within 0.1 logMAR) independent of age Gain ≥ -0.3 logMAR in 2/18 adult and 2/8 pediatric eyes Loss ≥-0.3 logMAR in 5/18 adult eyes	Blue Pediatric median – 5.85 dB Adult median -1 dB	Blue at 12 mo Pediatric median -18.98 dB Adult median – 8.95 dB	Mean CRT 165.87 µm ± 26.26 at BL, and 157.69 µm ± 30.3 at 12 mo; LLVA mean improvement −1.05 logMAR; all pediatric eyes had GVF III4e at BL, post VN improvement up to 50 %; No adult eye GVF III4e pre and post VN; DA-2CTP 2-color threshold perimetry significantly improved in pediatric eyes; new CRA in 50 % (42 % at injection site, 42 % central, 42 % peripheral); no correlation of CRA with change in FST at 12 mo

AEs adverse events of special interest; BCVA best corrected visual acuity; BL base line; CFT central foveal thickness; CRT central retinal thickness; CST central subfield thickness; CRA chorioretinal atrophies; DAC dark adapted campimetry, DA-2CTP dark adapted two color threshold perimetry; dB decibel; dpt diopter; EOSRD early onset severe retinal degeneration; FU follow-up; FST dark adapted Fullfield Light Stimulus Threshold; GVF Goldmann Visual Field; HM hand movements; LCA Leber congenital Amaurosis; LP light perception; logMAR logarithm of minimal angle of resolution visual acuity; LLVA low luminance visual acuity; mo months; ND no data; RP retinitis pigmentosa; VN voretigene neparvovec; y years

§ see also several later publications including [36] describing CRAs in 13 eyes/8 patients (including the 5 patients in [42]).

# includes the patients/eyes described in [42] and [36]

* includes cases reported by [27, 36, 42 ]

$ Includes cases reported by [13]

### Visual field changes

The effects on Goldmann visual field changes (as measured with Goldmann kinetic perimetry) are highly variable in the different reports (Table 2). In very advanced cases, little or no improvement was documented compared to earlier stages of the disease, i. e. generally speaking younger patients, where significant improvements were seen, probably related to better preserved outer retinal structures and underlying RPE, but not necessarily related to the location of the bleb at the end of the procedure.

### Dark-adapted chromatic fullfield light stimulus sensitivity (DA-cFST)

In most patients, regardless of age, there was a statistically significant change in retinal sensitivity, i. e. the patients were able to see the stimulus at lower light levels after VN. However, there was a tendency for younger patients to show more improvement. The DA-cFST with blue and red light stimuli allows estimating the effect on cone versus rod vision without providing information on spatial resolution. Briefly, the test is performed as follows: After pupil dilation and 45 min of dark adaptation, blue, red and white light flashes of 200 ms duration are presented with a Colordome full-field stimulator (Diagnosys LLC, Littleton, MA, USA, or equivalent). Each test takes 2 to 3 min. During the test, the EspionTM software uses a proprietary probability density function to automatically determine the threshold. In the FST protocol, the 0 dB baseline is defined as 0.1 cd/m^2^ for all three colours. The baseline luminance for the first trial is typically chosen to be at least 10 dB dimmer than the subject’s expected threshold to avoid light-induced rod desensitisation. A short break between sensitivity measurements avoids fatigue. A gain or loss of at least 10 dB is considered meaningful. A comparison of the thresholds for blue and red indicates whether the stimulus is perceived by rods or cones. [26].

### Two-color-threshold perimetry (2CTP)

**Figure 1:**
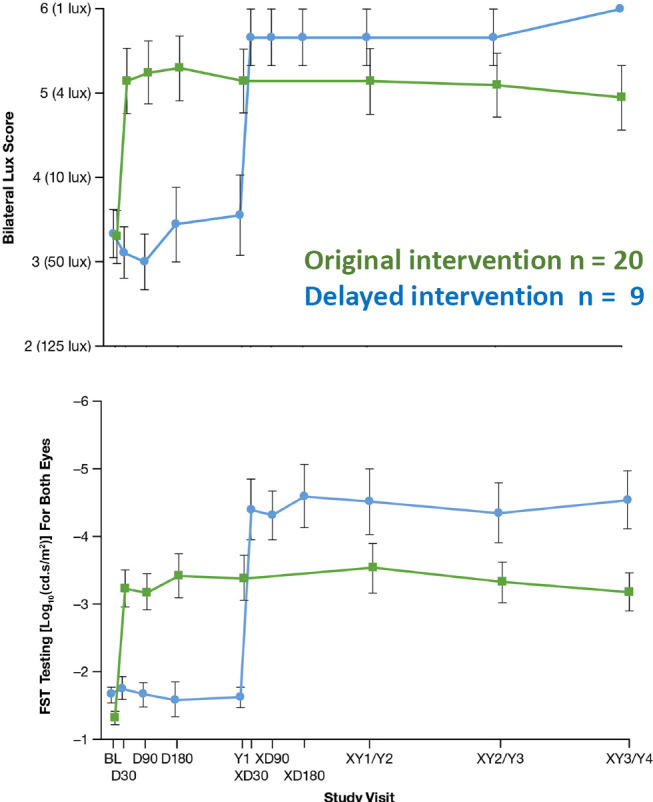
Comparison of multiluminance mobility test (MLMT)[7] and Fullfield Stimulus Light Test (FST) results up to 4 years after treatment with Voretigene Neparvovec in a phase 3 study. Modified after Maguire 2021 [29].

2CTP after dark adaptation measures retinal sensitivity to blue and red light at defined retinal locations. Similar to DA-cFST, it allows assessment of the more sensitive photoreceptor pathway by comparing blue and red thresholds. VN therapy showed significant improvements in rod function especially in younger patients [20, 26]. Unlike DA-cFST, 2CTP provides insight into spatially resolved changes in retinal sensitivity. 2CTP is not part of routine clinical practice but available in some highly specialized centers. The results compare well with the MLMT data [6], but the measurements are similarly time consuming. As mentioned above, DA-cFST is not a perfect substitute, as it is only a global test without spatial resolution and therefore does not efficiently delineate changes/enlargements of visual fields at low luminance levels (fig. 1 modified from [31]). However, in routine clinical practice, the DA-cFST is a very useful test and should be available in all centres where VN therapy is offered. In a survey conducted in 2021 by the European Vision Research Clinical Research network EVICR.net, 15 out of 26 centres following RPE65-IRD patients reported that they did not perform DA-FST [27], despite its importance in documenting detailed treatment effects on cones and rods, respectively, following VN therapy.

### New or accelerated chorioretinal atrophy as serious adverse effects

**Figure 2:**
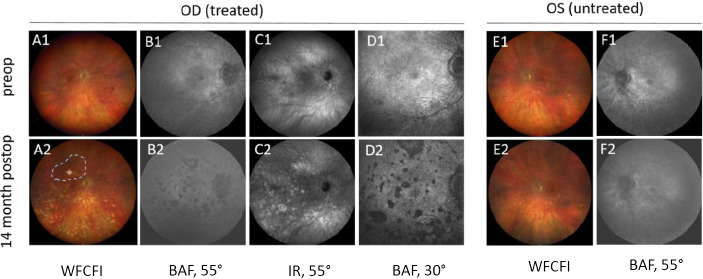
Accelerated retinal degeneration right eye after treatment with Voretigene Neparvovec. Non treated left eye stable during follow-up. Shown are widefield color images, blue light fundus autofluorescence images, and infrared images (right eye only). Modified after [26]. OD right eye, OS left eye.

Chorioretinal atrophy (CRA) inside and outside the bleb in up to 50 % and more of treated eyes (fig. 2), as well as inflammation, subretinal haemorrhage, subretinal neovascularization, subretinal deposits in young patients, and paracentral scotomas have recently been reported as a potential complication of VN therapy. [9, 12, 13, 18, 19, 25, 26, 36, 40]. Changes in retinal thickness have also been reported using optical coherence tomography (Table 2) [26]. CRA was not assessed in the phase 1 to 3 clinical trials. CRA represents irreversible photoreceptor loss associated with irreversible RPE atrophy. It has been suggested that CRA may be more common in myopic eyes and younger patients [13], but this has not been subsequently confirmed. An immunological or inflammatory response to the AAV capsid or gene product may also play a role in the development of atrophy [4, 5]. A recent paper speculated that empty capsids may cause adverse effects [3]. There is currently an open debate about the possible functional effects of CRA [42], with some claiming a higher improvement in DA-FST in the presence of CRA [42], which has not been confirmed by others [26, 32]. The effect on central visual function depends on the location and growth rate of the CRA. Four types were identified in 27 eyes treated in 5 US centres: (1) at the injection site = “touchdown”, (2) nummular (i. e. patchy CRAs) in the peripheral retina, (3) perifoveal in the bleb area, and (4) mixed forms [2]. The total number of eyes treated in these 5 centres was 187, resulting in an overall prevalence of CRA of 14.4 % in this particular study. The observed growth rates increased from type 1 to type 4. Perifoveal CRA may cause paracentral visual field defects and loss of BCVA, as reported by some authors (Table 2). In addition to the clear acceleration of disease in some patients following VN therapy, it is important to remember that already in 2013, progressive degeneration in both treated and untreated areas was reported in the nine patients studied, while improved retinal sensitivity persisted, albeit to varying degree [8]. CRA is controversial, both in terms of its underlying causes and its functional consequences. Further data are needed to draw conclusions. Modification of the surgery to apply VN may reduce the risk of touch-down CRA such as peeling of the internal limiting membrane at the site of the planned subretinal injection [10]. An interesting observation was made after VN therapy in the second eye of four patients previously treated with rAAV2-CB-hRPE65 as part of a gene augmentation clinical trial [20]. In 3 out of 4 eyes, areas of robust visual field improvement were followed by CRA 5 to 22 months after treatment with VN. The authors hypothesized that overexpression of normal RPE65 in eyes of patients with *RPE65-*IRD with a yet to define certain degree of diseased RPE may lead to an initial significant improvement of function followed by CRA. No atrophies were observed after the previous treatment with rAAV2-CB-hRPE65, where the treatment effects were in general much less pronounced suggesting a less effective vector.

### Conclusion and outlook

Post-marketing data from gene amplification therapy with VN (Luxturna™) in patients with *RPE65*-IRD confirm the benefit of increased visual performance under reduced light including low contrast vision. Long-term persistence of benefit for at least 4 to 5 years has been demonstrated in phase 1 to 3 clinical trials. Visual acuity and visual field results are variable and often do not reach statistical significance as variable results have also been observed in treatment-naïve patients [37]. Treating less advanced disease with a better preserved retina may be beneficial but patients with more advanced disease may also see some improvement. These beneficial effects need to be weighed against the acceleration of retinal degeneration that has been reported in up to 50 % of treated eyes post-marketing. Accelerated retinal degeneration can occur without functional loss as long as the fovea is not affected. Due to the high cost of VN therapy, the cost-benefit ratio must also be considered [17, 46]. Further studies should clarify the pathology of adverse effects and identify the therapeutic window in which patients benefit the most from therapy with the lowest risk of adverse effects. The introduction of VN therapy has clearly paved the way for gene therapy of IRDs. Other forms are currently under investigation and are expected to be approved in the near future – a great perspective for patients with inherited blinding diseases for which no causal treatment is yet available.
